# Green synthesis of ZnO-Zn-MOF/bacterial nanocellulose for ultra oxidative desulfurization of actual diesel fuel

**DOI:** 10.1038/s41598-025-14377-2

**Published:** 2025-08-10

**Authors:** Aya M. Matloob, Ola E. A. Al-Hagar, Deyaa Abol-Fotouh, Heba M. Salem

**Affiliations:** 1 Refining department, Egyptian Petroleum Research Institute (EPRI), Naser City, Cairo, 11727 Egypt; 2 Plant Research Department, Nuclear Research Center, Egyptian Atomic Energy Authority (EAEA), Cairo, 13759 Egypt; 3 Advanced Technology and New Materials Research Institute (ATNMRI), City of Scientific Research and Technological Applications (SRTA-City), 21934 Alexandria, Egypt

**Keywords:** Biodegradable catalyst, MOF, Bacterial nanocellulose, RGO, Zinc oxide, Oxidative desulfurization, Diesel oil, Biomaterials, Materials for energy and catalysis, Nanoscale materials

## Abstract

The oxidative desulfurization (ODS) process is a promising approach to reduce sulfur content in fossil fuels using efficient oxidant catalysts such as metal-organic frameworks (MOFs) and its composites. In this study, we aimed to exploit the amazing physicochemical properties of the naturally green polymer bacterial nanocellulose (BNC) as a platform for the ODS of actual diesel fuel. The methodology relied on a green one-pot solvothermal route to synthesize a ZnO-Zn-MOF/BNC nanocomposite. Owing to its 3D porous structure and richness of hydroxyl group, BNC served as a dispersing scaffold for growing the ZnO-Zn-MOF on its nanofibers, improved metal dispersion, and reduced catalyst aggregation. The prepared catalysts were thoroughly characterized using XRD, FTIR, SEM, TEM, and EDX. Moreover, BET analysis has interestingly revealed a morphological shift toward larger pores and enhanced interconnectivity, improving mass transfer and accessibility to active sites. Afterward, the ODS performance of the resulting ZnO-Zn-MOF/BNC nanocomposite was compared to the ZnO-Zn-MOF and ZnO-Zn-MOF/reduced graphene oxide (rGO) counterparts. Additionally, the impact of catalyst dosage, oxidant-to-sulfur molar ratio, extractant-to-oil ratio, reaction time, and temperature on the ODS efficiency of real diesel fuel was systematically investigated. Under optimized conditions 70 °C; O/S molar ratio of 8; catalyst dosage of 200 mg; solvent-to-oil ratio of 1.5:1; and 60 min reaction time, the ZnO-Zn-MOF/BNC catalyst attained 98.5% sulfur removal efficiency. Additionally, the catalyst maintained most of its structural integrity and catalytic activity over five successive cycles, demonstrating high resilience and potential for further in-depth studies to assess its viability for large-scale use as a green desulfurization toolkit.

## Introduction

Despite the global shift toward alternative energy sources like biofuels and hydrogen, fossil fuels are projected to remain a dominant part of the energy mix. According to the International Energy Agency, by 2040, global energy and fossil fuel demand is expected to rise by approximately 30%, underscoring the continued reliance on conventional fuels in the foreseeable future^1,2^. The strict regulations towards sulfur content that cannot exceed more than 15 ppm is one of the greatest difficulties in fulfilling the global demands on petroleum oil^3,4^.

The oxidative desulfurization (ODS) technique has also gained large interest as an alternative to hydrodesulfurization (HDS) due to its comparatively low cost, mild condition, and simplicity^5–8^. In ODS reaction, there are two steps involved in the process: the conversion of sulfur-containing compounds into their respective sulfoxides and sulfones, followed by extraction of these compounds using extractant^9–11^. The oxidation process requires oxidizing agent such as H_2_O_2_ which is considered a green oxidant with acidic nature^12^. Several solid catalysts had been examined for ODS reaction including heteropoly acid, either alone or supported^13^and metal oxides, i.e. oxides of (Mo, Mn, Sn, Fe, Co, Zn)^6,14^boron nitride^15^reduced graphene oxide (rGO)^16^activated carbon^17^zeolite^18^and metal–organic frameworks^19^.

Metal–organic frameworks (MOFs) are highly porous materials with potential applications in catalysis, sensors, drug delivery, gas separation, and storage^7,20–22^. These compounds are synthesized by special organizing of organic ligands and metal atomic cluster in special manner. Different synthesis methods have been developed for MOFs, including ultrasonic synthesis and micro-emulsion technology^23^. Additionally, MOFs are a type of macromolecule with a crystal structure and attaining significant porosity. This research field has rapidly developed in the past two decades^24^. MOFs can immobilize active functional materials and manufacture highly controllable nanostructures as carriers, providing new materials for many catalytic applications.

A variety of special characteristics of MOFs make them ideal for catalytic applications. Their remarkably large surface areas, which frequently surpass 3000 m^2^/g offer a wealth of active sites for catalytic reactions. MOFs’ catalytic effectiveness is increased by the selective adsorption and diffusion of reactants made possible by their adjustable pore sizes and shapes^25^. Furthermore, by altering the metal nodes or organic linkers, the framework composition can be changed, allowing materials with particular chemical functionalities to be designed for particular reactions. Additionally, a lot of MOFs exhibit strong chemical and thermal stability, which is crucial for real-world uses, especially in hostile reaction environments^26^. Additionally, they can be functionalized with different metals or active sites, which immobilizes catalysts and increase catalytic activity even more. These characteristics make MOFs appealing options for a variety of uses, such as gas separation, storage, and catalysis, especially in procedures like oxidative desulfurization^27^.

Among zeolitic imidazolate frameworks (ZIFs), Zn-BDC (zinc 1,4-benzenedicarboxylate) is a notable material because of its exceptional porosity, high specific surface area, and superior chemical stability. Because of these characteristics, Zn-BDC is a desirable option for a number of catalysis-related applications^28^. The customizable pore structure permits the selective diffusion of reactants, and the high surface area offers a large number of active sites for catalytic processes. Zn-BDC is appropriate for a variety of catalytic processes, such as oxidation, and desulfurization reactions, due to its chemical stability under various reaction conditions, which guarantees constant performance^29^.

Bacterial nanocellulose (BNC) recently came out as an eco-friendly polymer with surpassing physical properties. Several microbial strains were reported to produce BNC that has the same chemical composition as plant cellulose, where the structural unit is the firmly packed glucan chains resulting from the ꞵ-(1,4) cross-linking between glucose residues. Subsequently, this grants the overall BNC structure with inert chemical state which minimizes its direct reactivity with other species and maximizes its stability versus most organic solvents^30,31^.

BNC is produced in the form of nanofibers interwoven in a three-dimensional web, which endows the overall final structure with high surface area and nanoporous system^32^. Furthermore, the fiber diameter is about 100 times smaller than plant cellulose fibers and its Young’s Modulus is comparable to Kevlar^®^^33^. Additionally, BNC is naturally produced as hydrogel, which is rich in hydroxyl groups that allow chemical modification and conjugation with a broad range of materials. The resulting BNC composites find their ways to agglomerate applications such as biomedicine^34^drug delivery^35^cosmetics^36^biosensors^37^and energy generation modules^38,39^.

Bio-based polymers, such as BNC, have been widely adopted to remove hazards from ecological elements, such as wastewater, because they have no detrimental impact on the environment^40^. In contrast, the studies employed these sustainable polymers in treating fossil oil to reduce their sulfur compounds, such as benzothiophene and dibenzothiophene, which are still scarce. In one unique study, Rizzo and her coworkers have reported that the ionic liquid gels of cellulose and chitosan can be competitive to the common adsorptive materials to remove sulfur compounds from fossil fuels, not only for their high removal efficiency but for their re-usability potentiality as well^41^.

The integration of ZnO-Zn-MOF with BNC offers significant advantages, enhancing both the performance and sustainability of the catalyst in oxidative desulfurization (ODS). BNC, being a biodegradable and renewable material, provides a robust and eco-friendly platform that prevents particle agglomeration, increases dispersion, and ensuring better access to active sites. Moreover, BNC improves the thermal and chemical durability of the composite, endowing more tolerance for reaction conditions, and its low-cost production from renewable sources contributes to the economic feasibility of the catalyst. Furthermore, the potentiality of in situ or *ex situ* shaping BNC as porous membranes, spheres, or even macroparticles facilitates the treatment and post-treatment procedures, where the collection of the composite for re-using becomes more attainable^42^. These attributes make the ZnO-Zn-MOF/BNC composite a significant candidate for industrial desulfurization applications.

In this study, we developed a novel ZnO-Zn-based terephthalate framework and an innovative ZnO-Zn-MOF/BNC composite using an eco-friendly solvothermal synthesis method, emphasizing sustainability. These materials underwent characterization to analyze their structural and functional properties. Subsequently, we examined their potential for removing sulfur compounds from fossil fuel *via* oxidative process. The work plan included the ZnO-Zn-MOF/rGO composite to compare the physical characteristics and desulfurization performance. Various parameters, including nano catalyst dosage, oxidant ratio, reaction time, extractant-to-diesel ratio, and temperature, were evaluated for their impact on ODS efficiency. The physical and chemical properties of diesel oil before and after treatment with ZnO-Zn-MOF/BNC were also analyzed.

The principal objective of this study is to report for the first time, to the best of our knowledge, the feasibility of using bacterial nanocellulose (BNC) in desulfurization of fossil fuels through an eco-friendly synthesis strategy.

## Experimental

### Materials

Graphite, of Zn(NO_3_)_2_⋅6H_2_O, NaOH, terephthalic acid (H_2_BDC), triethylamine, acetonitrile, hydrogen peroxide, acetic acid, and dimethylformamide (DMF), ethanol, were purchased from Sigma Aldrich. Diesel oil (1200 ppm of sulfur) was obtained by the Cairo oil refining company.

### Methodology

#### Synthesis of bacterial nanocellulose (BNC)

Bacterial nanocellulose was synthesized by the strain *Komagataeibacter hansenii* KO28-05D. The strain was locally isolated and identified at the facilities of Microbiology laboratory, Plant Research Dept., Nuclear Research Center, EAEA^32^. To enhance its BNC productivity, the strain was treated with dual doses of^57^Co gamma irradiation (0.5 kGy, twice) *via* irradiator (MC20, Russia) through 0.77 kGy/h dose rate^43^. The irradiation procedures were conducted at The Cyclotron facility, Nuclear Research Center, EAEA.

### Catalyst preparation procedures

#### Synthesis of ZnO-Zn-MOF

To synthesize the composites, solutions of zinc nitrate hexahydrate (0.55 mol L^−1^) and sodium hydroxide (2.6 mol L^−1^) in ethanol were mixed. Subsequently, a solution of H_2_BDC (0.21 mol L^−1^) in ethanol and triethylamine was added under sonication. After sonication for 20 min, the precipitate was collected, washed with ethanol, and dried at 60 °C for 8 h^44^.

#### Synthesis of ZnO-Zn-MOF/BNC nanocomposite

Two sheets of BNC were treated with 1 N NaOH and stirred at 70 °C for 90 min, then the treated sheets were immersed in 0.55 mol L^−1^ of Zn(NO_3_)_2_.6H_2_O solution (12.32 g in 75 mL of ethanol) and stirring for 12 h. then 0.21 mol L^−1^ of H_2_BDC solution (6.64 g in 190 mL of ethanol and 10 mL of triethylamine) was added under sonication. After 20 min of sonication, the precipitate was collected, washed with ethanol, and dried at 60 °C for 8 h.

#### Synthesis of ZnO-Zn-MOF/rGO nanocomposite

To synthesize ZnO-Zn-MOF/rGO nanocomposite, 1 gram of ZnO-Zn-MOF was added to 0.5 g of graphite oxide dispersed in 50 mL of ethanol. The mixture was sonicated for 30 min and then stirred for 24 h. The resulting precipitate was collected, washed with ethanol, and dried at 60 °C for 8 h.

### Characterization of the synthesized catalysts

X-ray Powder Diffraction Analysis (XRD) was carried out using (Shimadzu XD-1) diffractometer with Cu Kα radiation (λ = 0.1542 nm), where the intensity data was collected at 25 °C in a 2θ range of 4–80° with a scan rate of 0.7°s^−1^. Fourier transformer infrared spectroscopy (FTIR) was adopted to investigate the function groups of the prepared samples, using (ATI Mattson 1001) in the wave number region of 400–4000 cm^−1^. The textural properties were calculated from N_2_ adsorption/desorption isotherms at liquid N_2_ temperature (− 196 °C) using (NOVA 3200 Unit, USA). Investigation by Scanning electron microscope (SEM) was scrutinized utilizing (JEOL JSM-6010LV, Japan). The transmission electron microscope (TEM) and energy dispersive X-ray (EDX) inspection was scrutinized using (TEM) (JEOL, JEM-2100, Japan) with 80 kV accelerating voltage. The mass fraction of sulfur was detected using energy-dispersive X-ray fluorescence spectrometry (XRF).

### Catalytic activity

The process of oxidative desulfurization (ODS) of diesel oil, containing 1200 ppm of sulfur, was conducted in a 50 mL flask. The procedure involved introducing 20 mL of diesel oil, a specific quantity of catalyst, and an oxidant into the flask, followed by stirring at 700 rpm for a predetermined duration. Subsequently, the catalyst was separated from the reaction mixture *via* centrifugation. Afterward, an extractant was added and allowed to interact for 30 min. The resulting mixture was gently transferred to a separator funnel and allowed to settle for approximately 10 min until no further increase in the extracted mixture was observed. The sulfur content was measured using XRF. The efficiency of ODS was calculated using the formula:

**Sulfur removal % = (S**_**i**_ **− S**_**f**_**)/S**_**i**_ **× 100**.

where S_i_ and S_f_ represent the initial and final sulfur concentrations in the diesel oil, respectively. Various parameters such as nano catalyst dosage (50–400 mg), oxidant ratio (2–10), reaction time (15–120 min), ratio of extractant to diesel oil (0.5:1–2:1), and reaction temperature (40–80 °C) were systematically evaluated to determine their impact on the efficiency of ODS of diesel oil.

## Results and discussion

### Catalyst synthesis and characterization

The irradiation of the strain *Komagataeibacter hansenii* KO28-05D by low doses of gamma ray seems to generate genetic mutation that enhances the BNC productivity, where the irradiating beam was reported to interact with the intracellular H_2_O molecules, producing free radical flood. These plenty of free radicals strike the key functional genes and cause gene devastation and recombination (i.e. genetic mutation). However, determining the exact mechanism beyond this is still under investigation^32,45^.

**Fig. 1:**
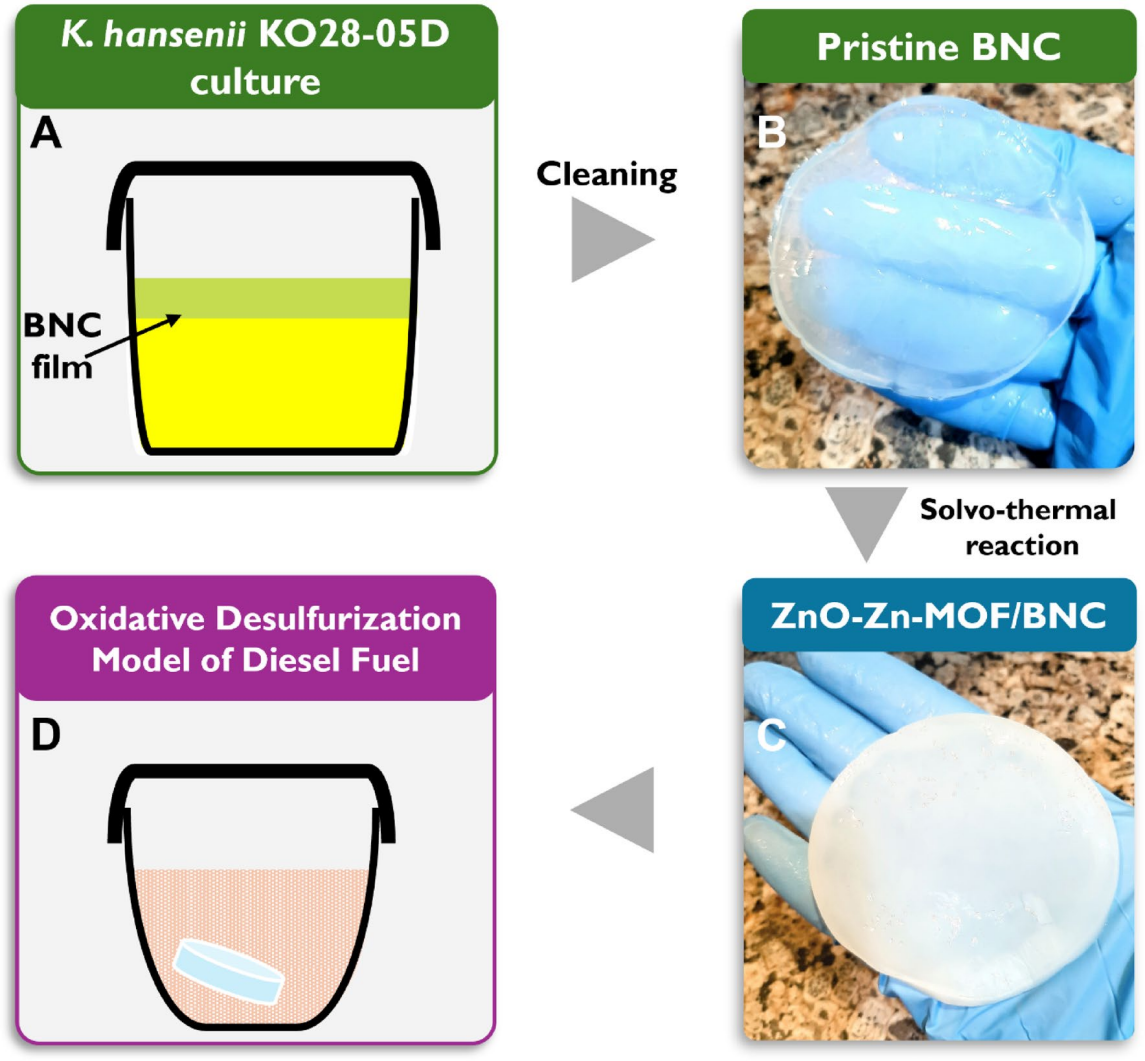
Schematic diagram summarizes the synthesis and examination of the ODS potential of the ZnO-Zn-MOF/BNC composite. Synthesis phase includes the biosynthesis of the BNC films by the bacterial strain *Komagataeibacter hansenii* KO28-05D (A); washing procedures to get the clean BNC film (B); the alcohol-based solvothermal reaction for producing the ZnO-Zn-MOF/BNC film (C); and evaluation of the ODS capability of the synthesized ZnO-Zn-MOF/BNC (D).

On the other hand, the all-water-based nature of the BNC biosynthesis elevates the eco-friendly footprints of the whole catalyst (Fig. 1A, B). Inclusion of BNC in the solvothermal reaction to raise the ZnO-Zn-MOF/BNC in situ seems to be effective strategy, where the transparent BNC hydrogel turned opaque and homogenously whitish in color (Fig. 1C). We assume that the porous system of the BNC structure allowed the reactants to easily disseminate through its 3D interconnected channels, where the reaction phases normally carried on. The synthesized nanocomposite underwent multiple characterization inspections before it was examined for its performance throughout the ODS process (Fig. 1D). The assurance of these speculations were carried out by the structural characterization.

**Fig. 2:**
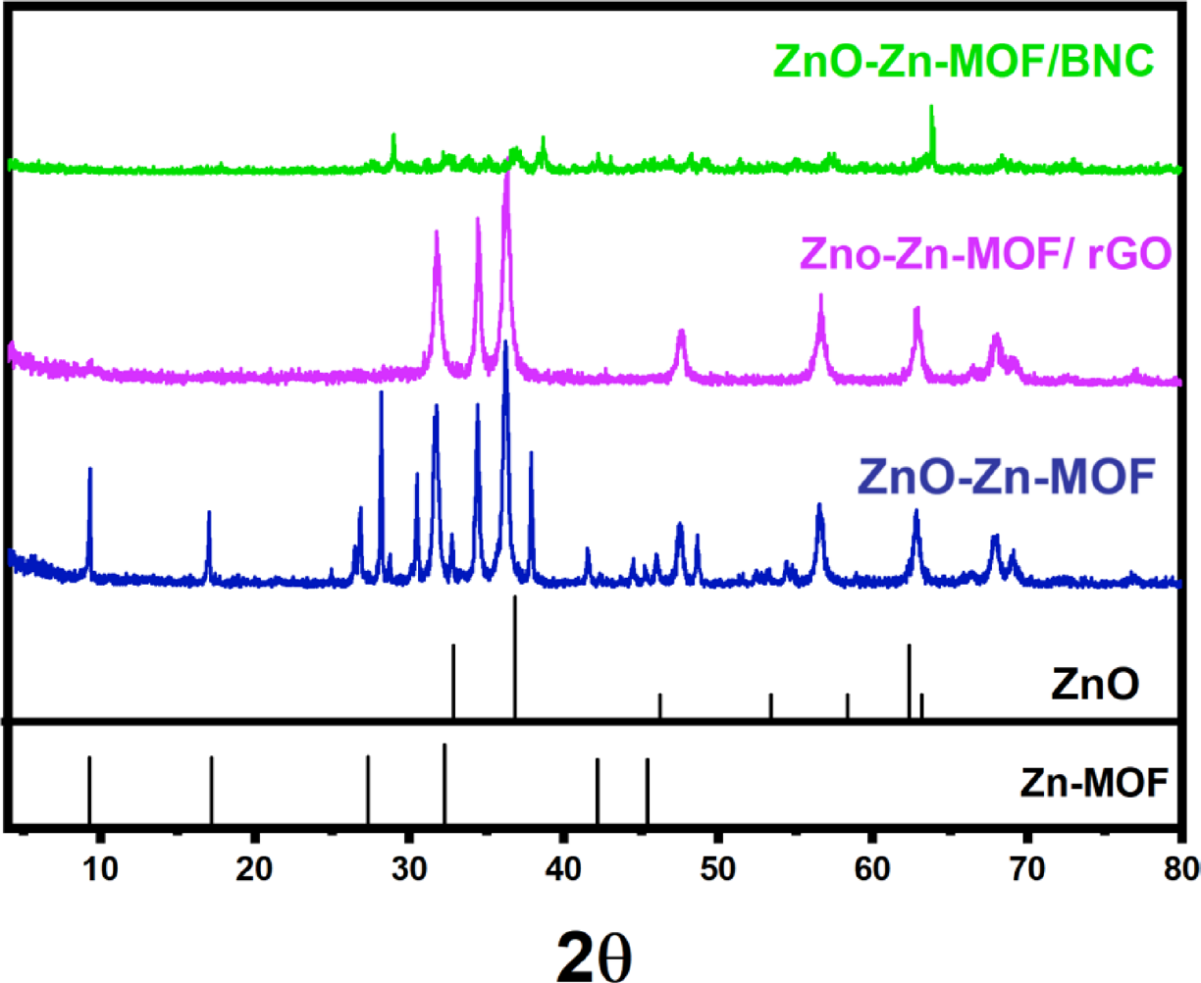
XRD patterns of the ZnO-Zn-MOF, ZnO-Zn-MOF/rGO, and ZnO-Zn-MOF/BNC nanocomposites.

**Fig. 3:**
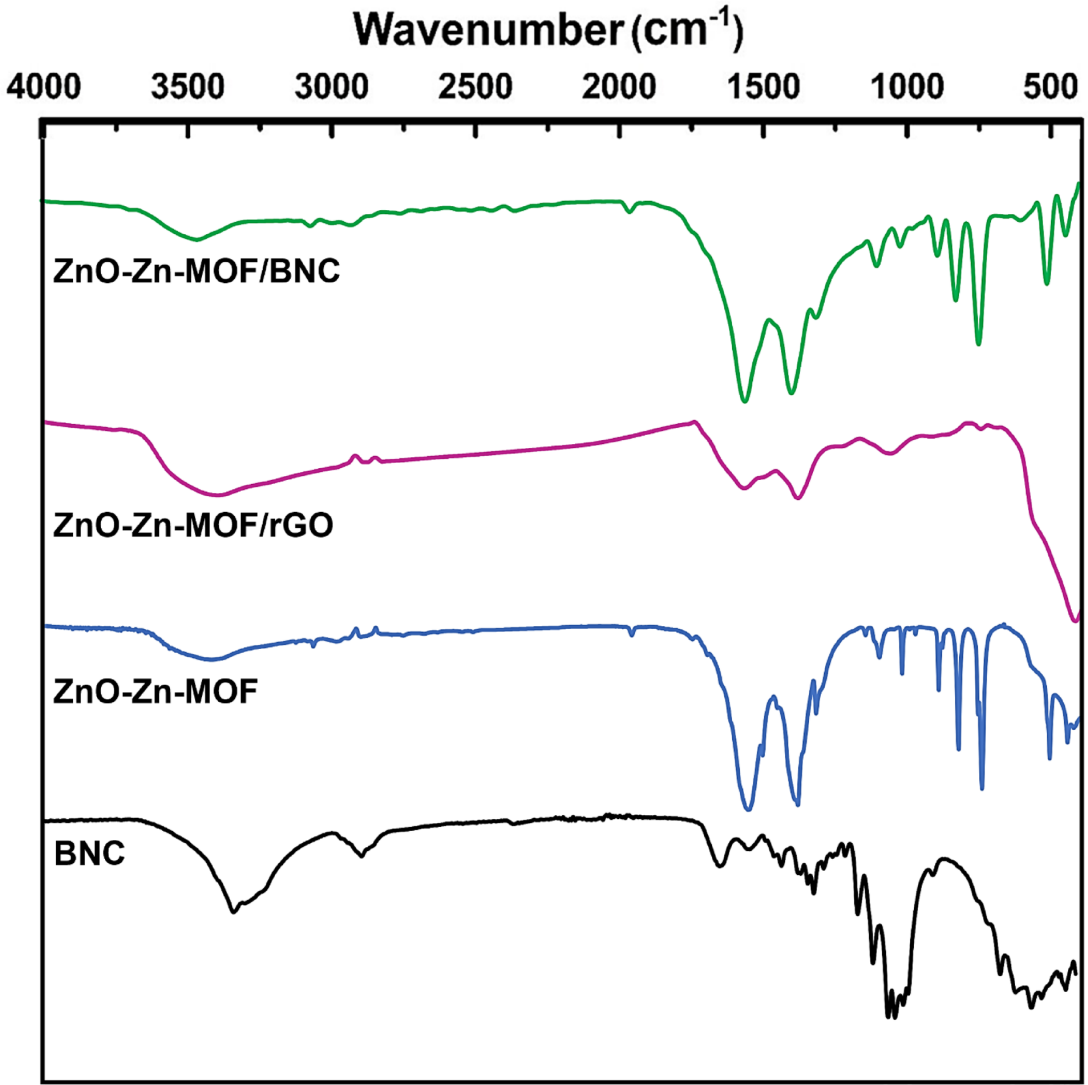
The FTIR of the BNC, ZnO-Zn-MOF, ZnO-Zn-MOF/rGO, and ZnO-Zn-MOF/BNC.

#### XRD analysis

The XRD diffraction patterns were recorded to clarify the phase purity and crystalline nature of the prepared samples (ZnO-Zn-MOF, ZnO-Zn-MOF/rGO, ZnO-Zn-MOF/BNC) nanocomposites. The XRD patterns of Zn-MOF in all the prepared samples were matched with the MOF-2. As pinpointed in Fig. 2, the experimental peak positions were at 2θ: 9.3°, 17.2°, 27.3°, 32.3°, 42.2°, and 45.4° relative to [200], [220], [400], [422], [600], [611] crystal plains for Zn-MOF, respectively^46,47^. Whilst the peaks at 31.3°, 34.3°, 36.3°, 48◦, 57°, 63.2°, 68.2°, and 69.2°.

are assigned to be associated with [100], [002], [101], [102], [110], [103], [112], and [201] crystalline surfaces of ZnO (JCPDS No. 75–0576)^48^.

In both ZnO-Zn-MOF/rGO and ZnO-Zn-MOF/BNC composites, the persistence of ZnO-Zn-MOF diffraction peaks confirms that the presence of rGO or BNC during synthesis does not interfere with the formation or crystallinity of the MOF structure. The simultaneous formation of ZnO and Zn-MOF can be attributed to a twin-phase interaction between Zn(OH)₂ and terephthalic acid under ultrasonic irradiation. Triethylamine plays a two-pronged role in the Zn-MOF synthesis reaction. Its strong basicity provides efficient deprotonation of terephthalic acid (H₂BDC), facilitating quicker coordination with Zn²⁺ ions and therefore inducing rapid nucleation of Zn-MOF crystals. Wherever BNC is involved, such deprotonation becomes localized within the nanofiber network, and this plays a role in spatially selective nucleation and subsequent crystal growth. This leads to a well-dispersed Zn-MOF structure embedded on the BNC framework.

Likewise. hydroxyl-rich and polar nature of BNC was proposed to attain steric and hydrogen-bonding interactions during MOF growth, stabilizing nucleated frameworks and preventing agglomeration. Simultaneously, ultrasonic cavitation was supposed to generate localized high-temperature regions that facilitate partial dehydration of Zn(OH)₂, leading to the formation of ZnO nanoparticles. The distribution of ZnO nanoparticles either on the external surface or within the pores of the MOF is regulated by several physicochemical parameters, including the porosity and surface energy of the growing MOF, the spatial effect of sonochemical cavitation, and molecular-level interactions with the BNC scaffold *via* hydrogen bonding and steric effects^49^.

Crystallite size calculations using the Scherrer equation found that the ZnO-Zn-MOF/BNC sample averages 26.56 nm, noticeably smaller than the 32.88 nm measured for the ZnO-Zn-MOF/rGO material. This may be due to that the incorporation of BNC may promote more homogeneous dispersion and limit crystal growth due to its nanofibrous 3D network, keeping particles evenly spread and curbing excessive coalescence. In addition, the ZnO-Zn-MOF/BNC pattern also showed broader and weaker diffraction peaks, specifically at 2θ values of approximately 31–36°, which exhibited greater microstrain and lattice disorder. These are plausibly associated with the flexible, hydroxyl-rich BNC surface enveloping each nanoparticle. More pointed peaks in the case of ZnO-Zn-MOF/rGO diffraction show greater domains and lower microstrain, which may be attributed to partial agglomeration on the two-dimensional rGO sheets. These results shed the lights on the role of the morphology of the support matrix in influencing the phase dispersion and crystal structure of hybrid catalysts^50^.

#### FTIR analysis

The functional groups of the prepared samples investigated by FTIR analysis and their spectra are presented in Fig. 3. The FTIR of the BNC sample elaborates a broad peak between wavelength 3000–3650 cm^−1^ assigned to -OH stretching which project from the glucan chains throughout the whole nanofibrils^51^. Whilst, the FTIR of the ZnO-Zn-MOF/BNC nano composite pinpoints shallow peak at the same wavelength range, suggesting involvement of this group in the ZnO-Zn-MOF/BNC conjugation.

Regarding the spectra of the ZnO-Zn-MOF and its composites, the bands emerged approximately at 3450 cm^−1^ display the –OH stretching group bound to ZnO, also the adsorption of water molecules during the preparation could contribute to the -OH band. The strong peaks at 1566 cm^−1^ represent the aromatic C = C in plane vibrations. The band at 1370 cm^−1^ assigned to the tricarboxylate coordination within the MOF framework. The bands at 1149 cm^−1^ correspond to C-C vibration. The peak at 1017 cm^−1^ is assigned to the vibration of ZnO–Zn fraction in the organic framework. The peaks at 876, 701, 526, and 450 cm^−1^ verified the absorption band characteristic of ZnO metal oxide^50,52,53^. The persistence and intensity of Zn–O and carboxylate coordination bands in the MOF/BNC spectrum indicates robust anchoring of MOFs to the biopolymer, confirming a stable organic-inorganic interfacial interaction.

On the other hand, the projection at 2901 cm^−1^ in the ZnO-Zn-MOF/rGO specimen is attributed to carbonyl groups (C = O) resulting from the reduction of rGO^54^. Additionally, the lower intensity of the peaks may be attributed to a partial overlapping between the rGO sheets and the MOF structure^21^.

#### BET analysis

**Fig. 4:**
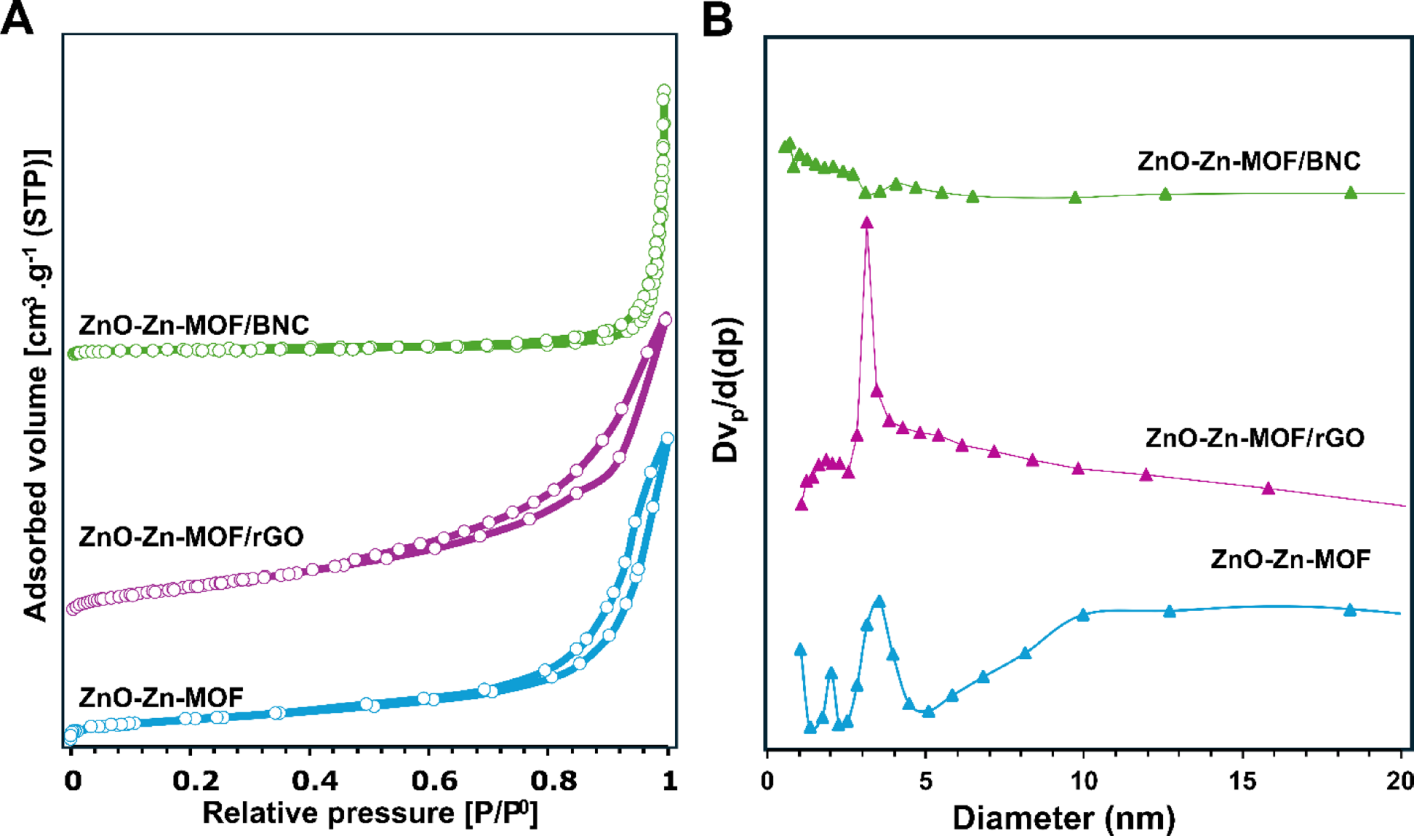
(A) N_2_ adsorption-desorption isotherms; and (B) pore size distribution of the prepared samples.

The BET surface area and pore structure of the synthesized materials were analyzed using N₂ adsorption–desorption isotherms (Fig. 4A). All samples exhibited characteristics of type-IV isotherms according to Brunauer’s classification, accompanied by a distinct hysteresis loop, indicating the presence of mesoporous structures^55^. The pristine ZnO-Zn-MOF composite exhibited a specific surface area of 38.1 m²/g, an average pore size of 8.5 nm, and a total pore volume of 0.1654 cm³/g. Upon incorporation of rGO and BNC, notable changes in the textural properties were observed. The surface areas of ZnO-Zn-MOF/rGO and ZnO-Zn-MOF/BNC were reduced to 35.57 and 4.437 m²/g, respectively, while their average pore sizes increased to 12.7 nm and 31.9 nm. The corresponding pore volumes also changed to 1.13 and 0.037 cm³/g, respectively.

The marked decrease in surface area for the ZnO-Zn-MOF/BNC composite suggests a potential constraint on mass transfer efficiency. However, this is counterbalanced by a substantial increase in average pore size (Fig. 4B), reflecting a morphological transition toward a more open and interconnected porous architecture. This shift is advantageous for catalytic processes, as it enhances the accessibility of active sites, particularly those located on external surfaces or at the interfacial regions between ZnO nanoparticles, MOF domains, and BNC fibers.

Furthermore, the relatively higher total pore volume of the ZnO-Zn-MOF/rGO composite and the open network facilitated by BNC promote effective reactant diffusion. The fibrous, hydroxyl-rich structure of BNC also plays a key role in dispersing the catalytic components and suppressing particle aggregation. This structural synergy ultimately contributes to improved catalytic activity, as illustrated in the application part by the enhanced performance of ZnO-Zn-MOF/BNC in oxidative desulfurization.

#### SEM investigation

**Fig. 5:**
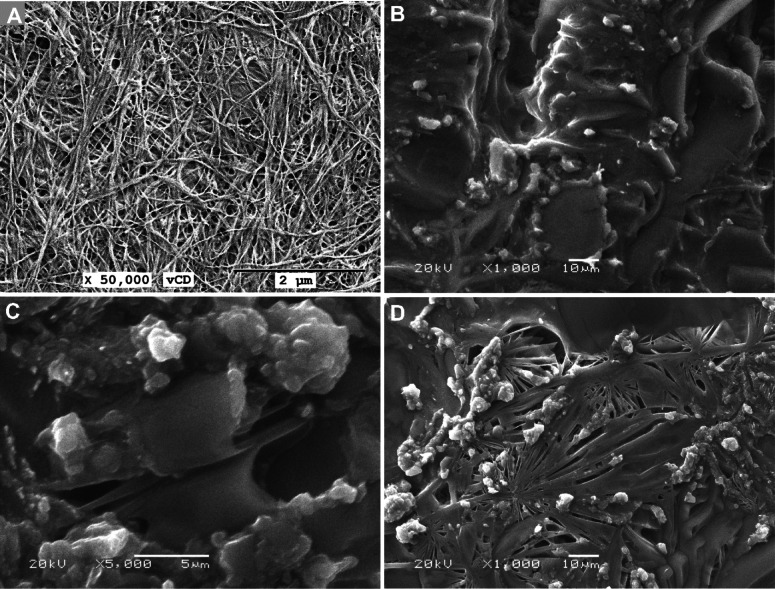
SEM images of (A) ZnO-Zn-MOF; (B) ZnO-Zn-MOF/rGO; and (C) ZnO-Zn-MOF/BNC.

Scanning Electron Microscope (SEM) analysis was used to study the microstructure of synthesized composites. Figure 5A displays the consistency of the ZnO-Zn-MOF sample, where the organic fraction vividly provides an irregular texture with minimal characteristic features. We can recognize some nanoparticles, with non-uniform size and shape, scattered throughout the organic fraction. A better characterization opportunity may be gained by the TEM inspection. Regarding the SEM of the ZnO-Zn-MOF/rGO and ZnO-Zn-MOF/BNC composites (Fig. 5B & C), the low featuring potentiality did not improve, except in the case of the ZnO-Zn-MOF/BNC imaging, where the distinctive interconnected nanofibrous web of the BNC has been recognized, where the ZnO-Zn-MOF particles were anchored to the BNC fibers (Fig. 5D).

#### TEM investigation

**Fig. 6:**
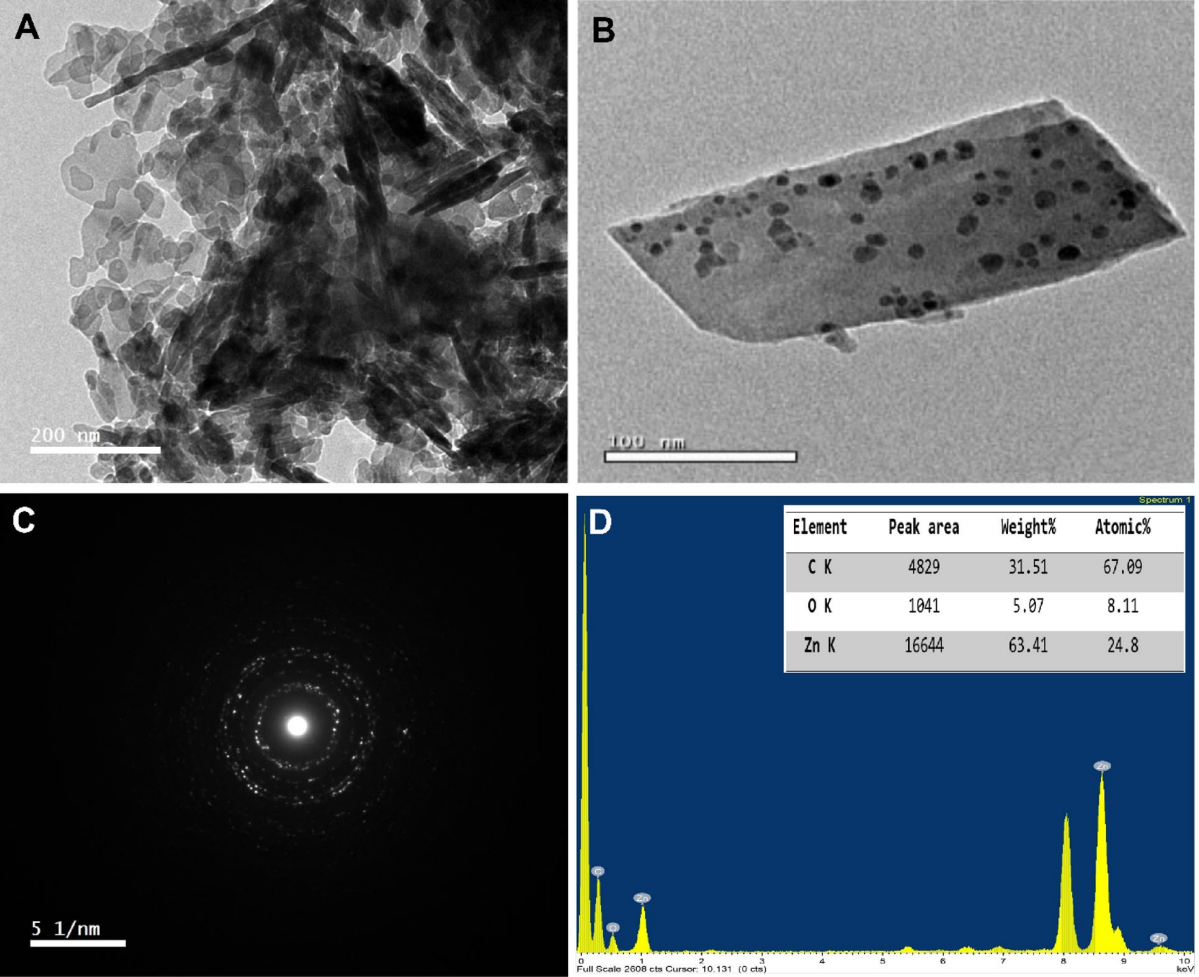
TEM image of (A) ZnO-Zn-MOF and (B) ZnO-Zn-MOF/rGO; (C) SAED pattern of ZnO-Zn-MOF; and (D) EDX analysis of ZnO-Zn-MOF.

Utilizing the TEM tool, the ZnO-Zn-MOF exhibited an overlapping nanotexture where non-uniform nanoparticles were clustered together (Fig. 6A). At higher magnification, these crystals displayed a variety of random geometries, including flat tetragonal flakes stacked atop one another. This can be attributed to the tetragonal crystal structure characteristic of the selected metal-organic framework, indicating anisotropic facets that expose more catalytically active Zn^2+^ centers; a significant feature for boosting sulfone desorption efficiency during the ODS process. The terephthalic acid ligand coordinates with Zn²⁺ ions through its dicarboxylic functional groups to form extended 2D square lattices^56,57^.

As shown in Fig. 6B, the prepared rGO displayed a uniform sheet-like structure, with Zn-MOF crystals well-dispersed across its surface. The rGO nanosheets acted as a physical support that effectively suppressed MOF aggregation, enabling better dispersion and enhancing surface accessibility of the composite. Pooresmaeil et al.. proposed that during the conjugation of zinc MOFs with rGO, Zn²⁺ ions coordinate with either the carboxyl groups of terephthalic acid or functional sites on rGO, facilitating MOF formation directly on the nanosheet surface^49^. In this study, no free Zn-MOF particles were observed detached from the rGO matrix, reinforcing the idea that the 2D rGO serves as an excellent substrate for MOF crystallization.

This structural integration is further supported by Fig. 6C, where the Selected-Area Electron Diffraction (SAED) pattern of ZnO-Zn-MOF presents multiple well-defined concentric rings, indicating the polycrystalline nature of the composite, consistent with the XRD analysis^58^. Additionally, EDX analysis (Fig. 6D) confirmed a high zinc content, with Zn accounting for more than 63 wt% of the total composition.

We propose that the dispersion behavior is strongly influenced by the role of triethylamine (TEA), which acts as a mild base during synthesis. TEA facilitates the deprotonation of H₂BDC, enabling rapid coordination with Zn²⁺ ions and promoting uniform nucleation^47^. Additionally, the hydroxyl-rich and spatially confined environment of BNC introduces steric hindrance and hydrogen bonding, both of which constrain crystal overgrowth and aggregation. As a result, the Zn-MOF domains remain small and well-distributed throughout the BNC matrix. This cooperative effect between TEA and the BNC scaffold not only supports controlled crystal growth but also enhances interfacial contact and accessibility of catalytic sites, contributing to the performance of the composite in desulfurization applications.

### Catalytic performance

#### Evaluation of desulfurization potential of ZnO-Zn-MOF and its nanocomposites

**Fig. 7:**
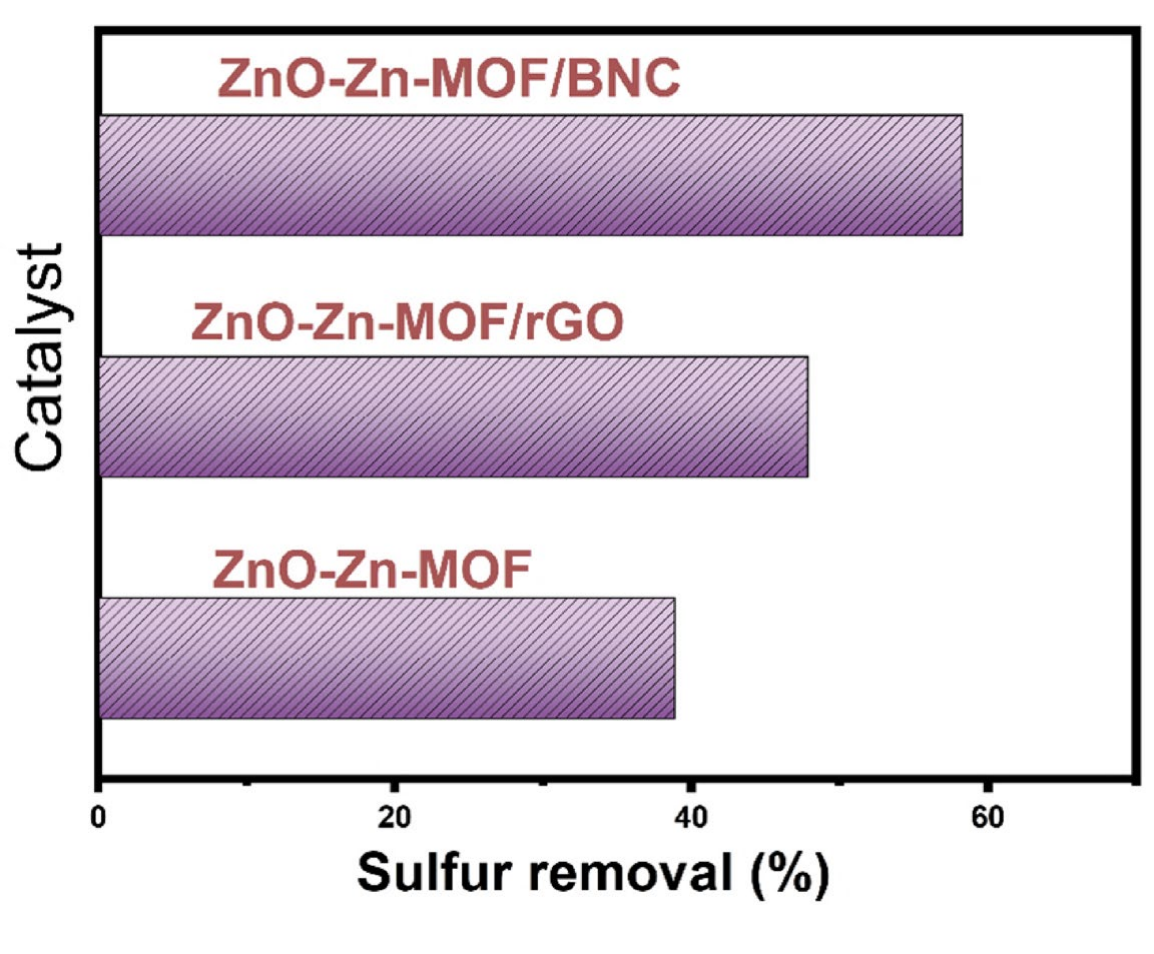
The influence of different types of catalysts on sulfur removal by the ODS reaction.

The oxidative desulfurization performance of the prepared catalysts ZnO-Zn-MOF,

ZnO-Zn-MOF/rGO, and ZnO-Zn-MOF/BNC was evaluated using 20 mL of diesel fuel containing 1200 ppm sulfur. Out of the three examined materials, the ZnO-Zn-MOF/BNC hybrid exhibited the highest sulfur removal efficiency, reaching 58.24%. Whilst, ZnO-Zn-MOF showed the lowest efficiency at 38.8%, while adding rGO to the ZnO-Zn-MOF improved the performance to 47.8% (Fig. 7). This performance can be attributed to the unique properties of BNC which facilitate rapid mass transfer of sulfur molecules and provide mechanical stability to the composite material. Additionally, BNC’s polar and hydroxyl-rich surface was proposed to contribute better interaction with oxidized sulfur species, favoring sulfone adsorption and thus improving desulfurization efficiency unlike hydrophobic materials such as reduced graphene oxide (rGO). Furthermore, the synergistic interaction between ZnO-Zn-MOF and BNC is suggested to contribute in a stronger adsorption affinity for sulfur compounds, enhancing the overall desulfurization efficiency of the ZnO-Zn-MOF/BNC nanocomposite.

**Fig. 8:**
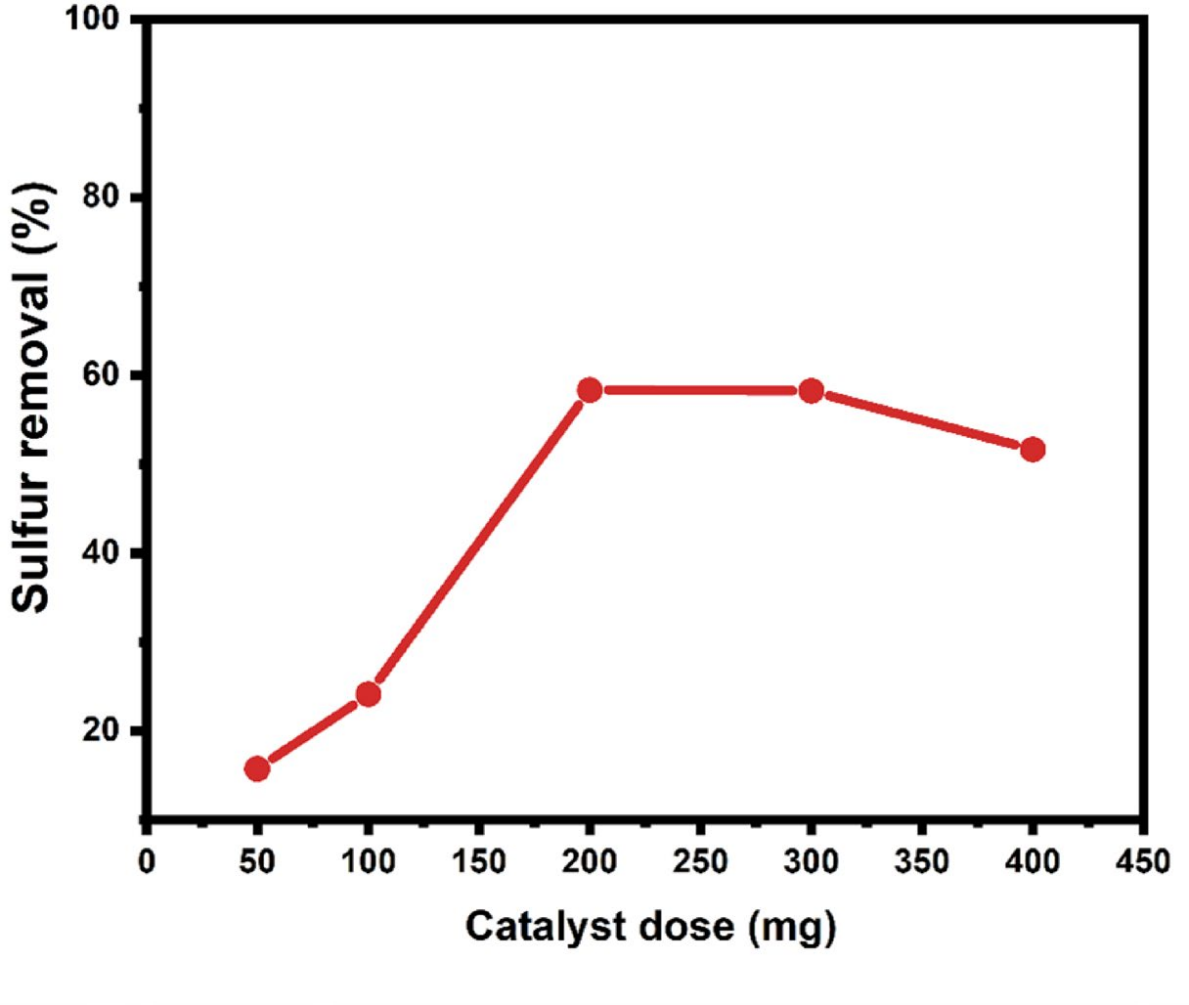
The influence of catalyst dose on sulfur removal by the ODS reaction.

**Fig. 9:**
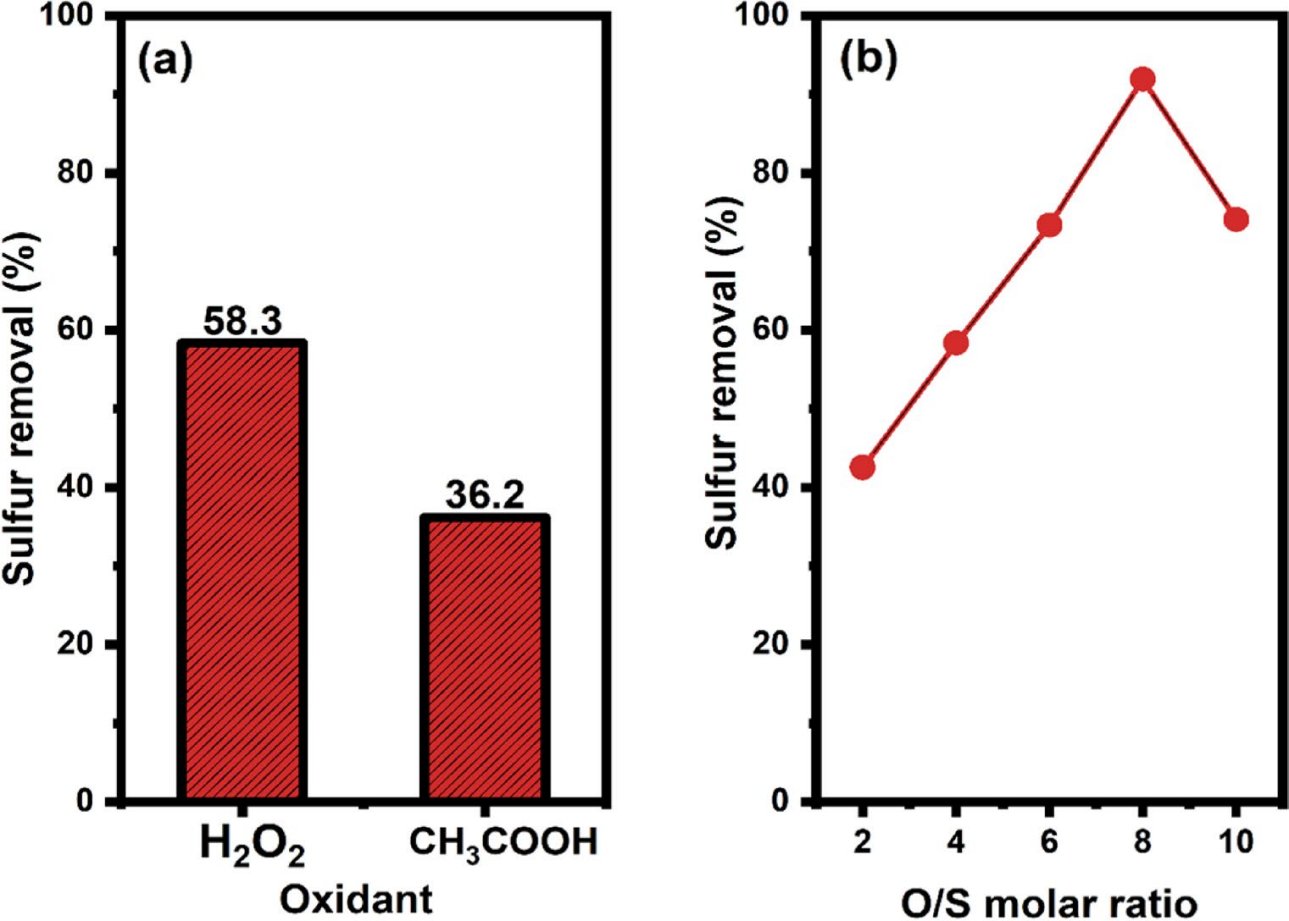
The influence of an oxidant type A), O/S B) on sulfur removal by. ZnO-Zn-MOF/BNC catalyst.

#### The influence of catalyst dose

The impact of ZnO-Zn-MOF/BNC catalyst dosage on desulfurization efficiency was investigated, considering its crucial importance for industrial applications. These investigations were conducted under reaction conditions, including 20 mL of diesel oil; an H_2_O_2_ to sulfur ratio (O/S) of 4; a 1:1 ratio of acetonitrile to diesel oil; and an oxidation reaction time of 60 min at 323 K. As depicted in Fig. 8, it is evident that increasing the ZnO-Zn-MOF/BNC catalyst dosage up to 200 mg notably enhances the percentage of sulfur removal. This enhancement is attributed to the rise in the number of active sites within the catalytic system^1^elevating the contact between sulfur molecules and the catalyst for oxidation. However, with the elevation of the ZnO-Zn-MOF/BNC concentration to 300 mg, no further improvement in sulfur removal was observed, indicating an equilibrium state between sulfur-containing diesel oil and active sites on the catalyst. Consequently, 200 mg was determined as the optimal ZnO-Zn-MOF/BNC catalyst dosage and was employed for subsequent investigations.

#### The influence of the oxidant

One of the critical factors determining the efficiency of the extractive oxidative desulfurization (EODS) is the selection of the oxidant. In this experiment, hydrogen peroxide and acetic acid were considered as potential oxidants. Each test involved consistent parameters: 20 mL of diesel oil; 200 mg of catalyst; an O/S ratio of 4; a 1:1 ratio of acetonitrile to diesel oil; and a 60-min oxidation reaction time at 323 K. As depicted in Fig. 9A, the oxidation reactivity was notably higher in the presence of H_2_O_2_ compared to acetic acid. Interestingly, H_2_O_2_ demonstrated greater efficacy in total sulfur removal, achieving an EODS efficiency of 58.3%, versus only about 36.2% accomplished by acetic acid. Consequently, H_2_O_2_ was chosen as the oxidant for all subsequent activity tests in this study. Figure 9B illustrates the investigation into the effect of varying amounts of H_2_O_2_ (represented as O/S molar ratios) over ZnO-Zn-MOF/BNC catalyst.

The study explored different O/S molar ratios of H_2_O_2_, including ratios of 2, 4, 6, 8, and 10. These experiments were conducted under consistent reaction conditions: 20 mL of diesel oil; 200 mg of catalyst; a 1:1 ratio of acetonitrile to diesel oil; and a 60-min oxidation reaction time at 323 K. According to the stoichiometry of the reaction, only two moles of H_2_O_2_ are required per a mole of sulfone formed. It is observed that sulfur removal significantly improves as the O/S molar ratio increases up to 8. The increase from an O/S molar ratio of 2 to 8 resulted in sulfur removal increasing from 42.5 to 91.9%. However, when the O/S molar ratio exceeded (10), sulfur removal decreased to almost 74%. This decline may be attributed to excessive hydrogen peroxide leading to H_2_O molecules occupying active sites, thereby reducing sulfur adsorption by the ZnO-Zn-MOF/BNC and hindering sulfur removal (%)^59^. Consequently, an O/S molar ratio of (8) was determined as the optimal ratio of H_2_O_2_ and was utilized for further investigations.

#### The influence of oxidation reaction time

**Fig. 10:**
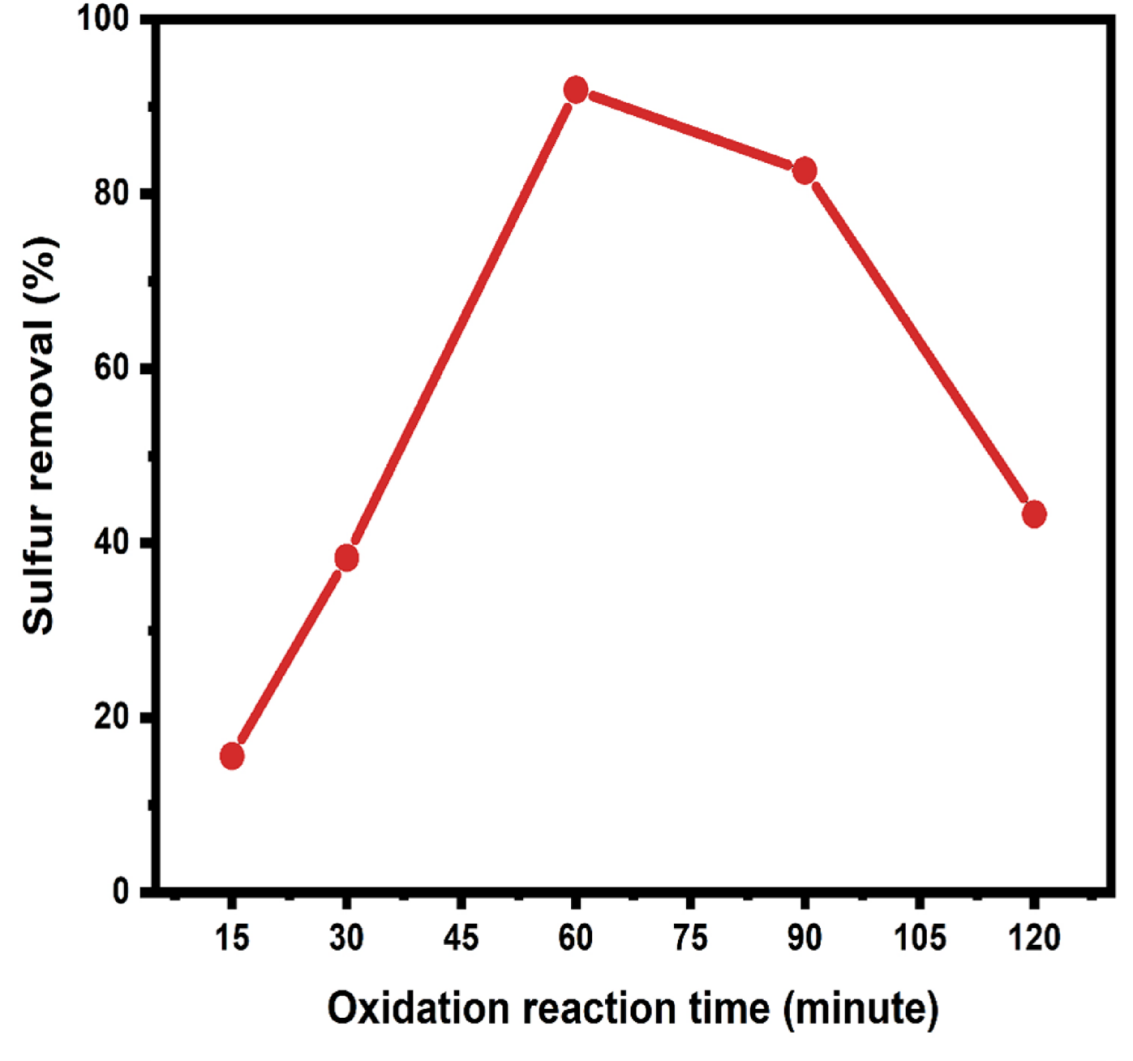
The influence of oxidation reaction time on sulfur removal by the ODS reaction.

The impact of oxidation reaction time on the efficiency of the EODS process was investigated by varying the reaction time from 15 to 120 min (refer to Fig. 10) while keeping all other process parameters constant, including 20 mL of diesel oil; 200 mg of catalyst; a 1:1 ratio of acetonitrile to diesel oil; and an O/S ratio of (8) at a temperature of 323 K. The results revealed that with an increase in reaction time from 15 to 60 min, there was a substantial rise in the percentage of sulfur removal, escalating from 15.6 to 91.3%. This enhancement may be explained by the extended contact duration between the catalyst and sulfur molecules, which facilitates their oxidation.

**Fig. 11:**
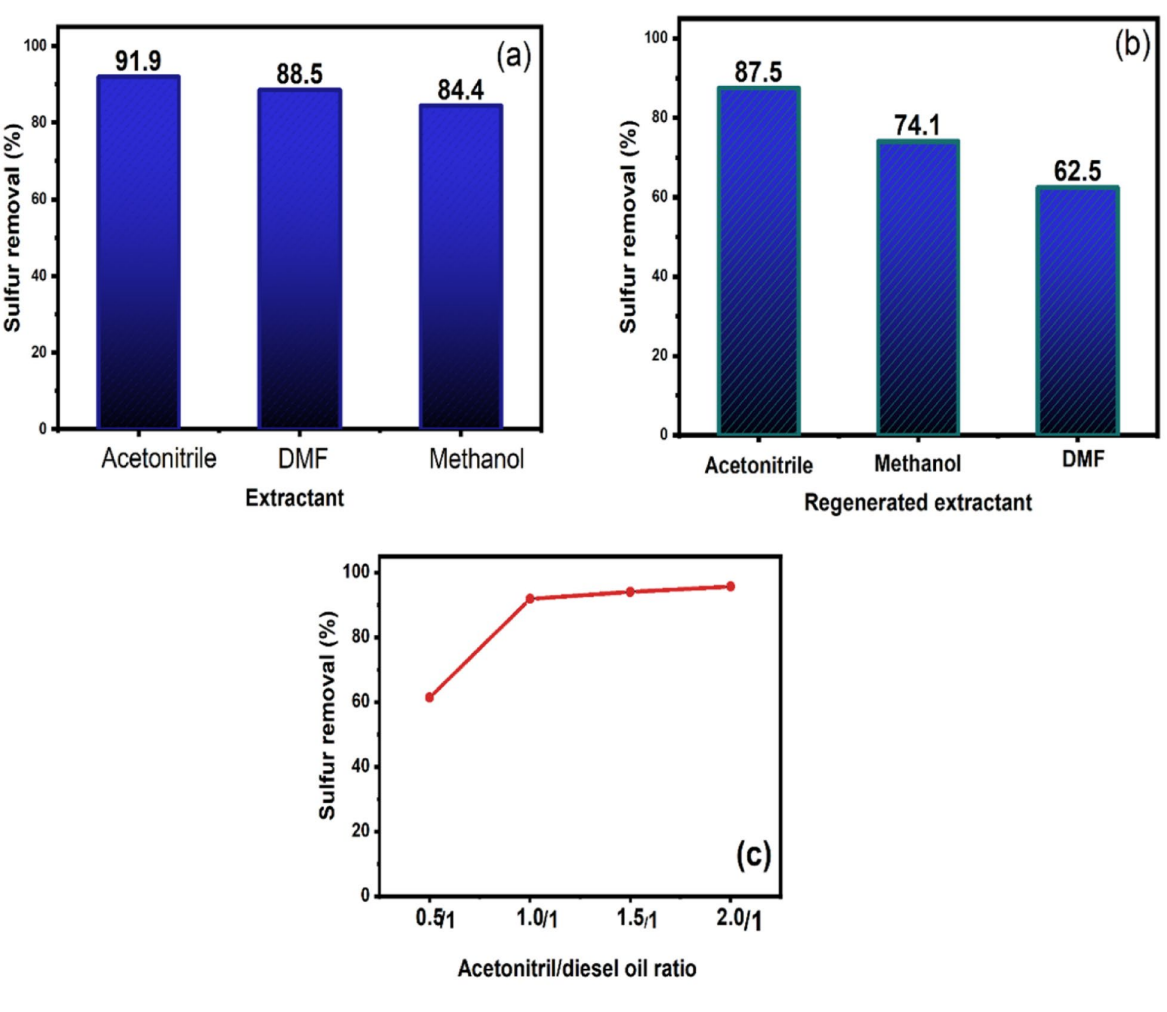
The influence of an extractant type A), regenerated extractant B), and the acetonitrile to diesel oil ratio C) on sulfur removal.

**Fig. 12:**
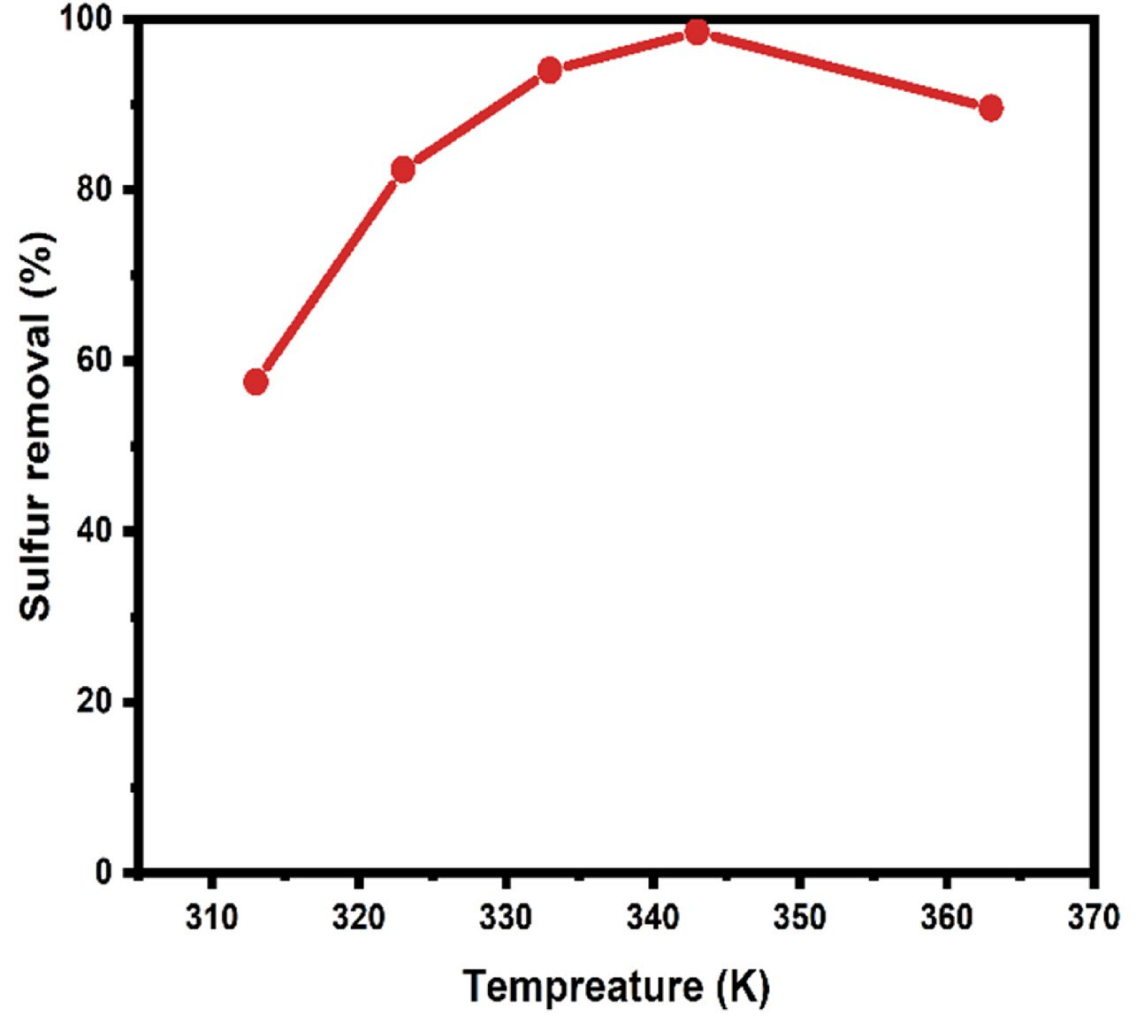
The impact of reaction temperature on sulfur removal by the ODS reaction.

However, after 60 min, there was a decline in sulfur elimination. We presume that the decline in activity after 60 min is due to H₂O₂ decomposition at 343 K, where the excessive thermal degradation reduces radical formation. Fenton-like reactions involving Zn²⁺ may play a secondary role, although this was not explicitly tested. Consequently, the 60 min span was determined as the optimal oxidation reaction time for this study, balancing enhanced sulfur removal with the avoidance of oxidant decomposition.

#### The influence of the extractant

Sulfur compounds in oil undergo conversion to sulfoxides and sulfones during oxidation, allowing for subsequent extraction using polar solvents. The efficacy of extractive oxidative desulfurization relies heavily on the solubility of these organosulfur compounds in the chosen extractant^6^. Therefore, selecting an appropriate solvent is critical for successful desulfurization. Methanol, dimethylformamide (DMF), and acetonitrile were employed as extraction solvents in this study due to their well-known effectiveness. These experiments were conducted under identical reaction conditions: 20 mL of diesel oil; 200 mg of catalyst; a 1.5:1 ratio of acetonitrile to diesel oil; and an O/S ratio of 8 at 323 K.

From the findings in Fig. 11 A, it is evident that acetonitrile was the most effective in extracting oxidized sulfur compounds from diesel oil. Subsequently, methanol, DMF, and acetonitrile were tested after regeneration through distillation to achieve economic benefits, as shown in Fig. 11B. Results indicated that acetonitrile exhibited the highest sulfur removal compared to other solvents. The decrease in sulfur removal from 91.5% for fresh acetonitrile to 87.5% when using regenerated acetonitrile represented only a 4% reduction, making it the best regenerated solvent.

Consequently, acetonitrile was chosen as the extraction solvent to leverage its high regeneration capability and superior desulfurization efficiency. Following this, the impact of the acetonitrile to diesel oil ratio on sulfur removal percentage was studied to determine the optimal ratio. As demonstrated in Fig. 11C, the sulfur removal percentage increased with the acetonitrile to diesel oil ratio. Specifically, the sulfur removal percentage increased from 61.4 to 91.9% as the acetonitrile to diesel oil ratio was adjusted from 0.5:1 to 1.5:1. The improvement was attributed to enhanced mass transfer across the polar-nonpolar interface between acetonitrile and diesel oil. While a slightly higher acetonitrile to diesel oil ratio of 2:1 led to a slight increase in desulfurization efficacy, the ratio of 1.5:1 was deemed preferable economically. Thus, a ratio of 1.5:1 was selected as the optimal acetonitrile to diesel oil ratio for this study.

#### The influence of reaction temperature

Temperature plays a crucial role in determining the catalytic efficiency of the ODS process^3^. To explore its impact, a series of experiments were conducted across a temperature range of 40 to 90 °C, under fixed reaction conditions: 20 mL of diesel oil (1200 ppm sulfur content); O/S molar ratio of 8; 200 mg of ZnO-Zn-MOF/BNC catalyst; a 1.5:1 acetonitrile-to-diesel ratio; and a reaction time of 60 min. As illustrated in Fig. 12, sulfur removal efficiency significantly increased with temperature, rising from 57.5% at 40 °C (313 K) to 98.5% at 70 °C (343 K). This enhancement is attributed to the temperature-dependent acceleration of oxidation reactions and improved desorption of the formed sulfone species from the catalyst’s active sites. At lower temperatures, strong sulfone adsorption can hinder access to reactive centers, limiting further oxidation of sulfur compounds.

The sulfur removal at 70 °C (343 K) reflects a balance between favorable reaction kinetics and thermodynamic constraints that govern both oxidant behavior and catalyst stability. From a kinetic viewpoint, increasing temperature typically enhances reaction rates by helping to overcome the activation energy barrier associated with oxidative desulfurization. At 70 °C, the thermal energy is sufficient to promote the generation of reactive oxygen species (ROS) from H₂O₂, particularly hydroxyl (•OH) and hydroperoxyl (HO₂•) radicals, without inducing excessive decomposition of the oxidant. These ROS are essential for effectively oxidizing refractory sulfur compounds in diesel. However, from a thermodynamic standpoint, hydrogen peroxide becomes increasingly unstable at temperatures above 70 °C. The enthalpy of its decomposition (∆H ≈ − 98 kJ/mol) drives rapid breakdown into water and oxygen, leading to a decline in the concentration of active radical species. As a result, sulfur removal efficiency plateaus or even declines at higher temperatures due to insufficient ROS availability.

Moreover, elevated temperatures were reported to cause undesirable changes in the assembly or features of the catalyst, such as structural degradation, phase transformation, sintering, and/or leaching^60^. Although the Zn-MOF framework is known to exhibit good thermal stability, localized heating effects, particularly under sonication or oxidative conditions, may lead to partial sintering or surface restructuring of ZnO domains^61^. These structural changes can reduce the available surface area or block pore networks, thereby impair mass transfer and limit access to active sites. Therefore, we presume that the temperature around 70 °C represents a critical threshold where the rates of radical formation, oxidant stability, and catalyst structural integrity are all favorably aligned. Exceeding this temperature degree is anticipated to disrupt the synergy between these factors, ultimately resulting in reduction of the ODS efficiency.

#### Physicochemical characterization of diesel oil

Table 1 displays the physical and chemical properties of the diesel oil before and after undergoing EODS treatment using ZnO-Zn-MOF/BNC under optimal conditions (20 mL of diesel oil (1200 ppm); O/S = 8; 200 mg of catalyst; acetonitrile/diesel oil = 1.5:1; and a 60 min oxidation reaction time at 343 K). Following EODS treatment, there is a significant reduction in the total sulfur content from 1200 ppm to 18.1 ppm. As depicted in Table 1, the physical properties of the diesel oil remained almost the same after the EODS process, suggesting that utilizing ZnO-Zn-MOF/BNC for desulfurization had no adverse effects on the original diesel oil’s physicochemical characteristics.

#### Catalyst reusability

**Fig. 13:**
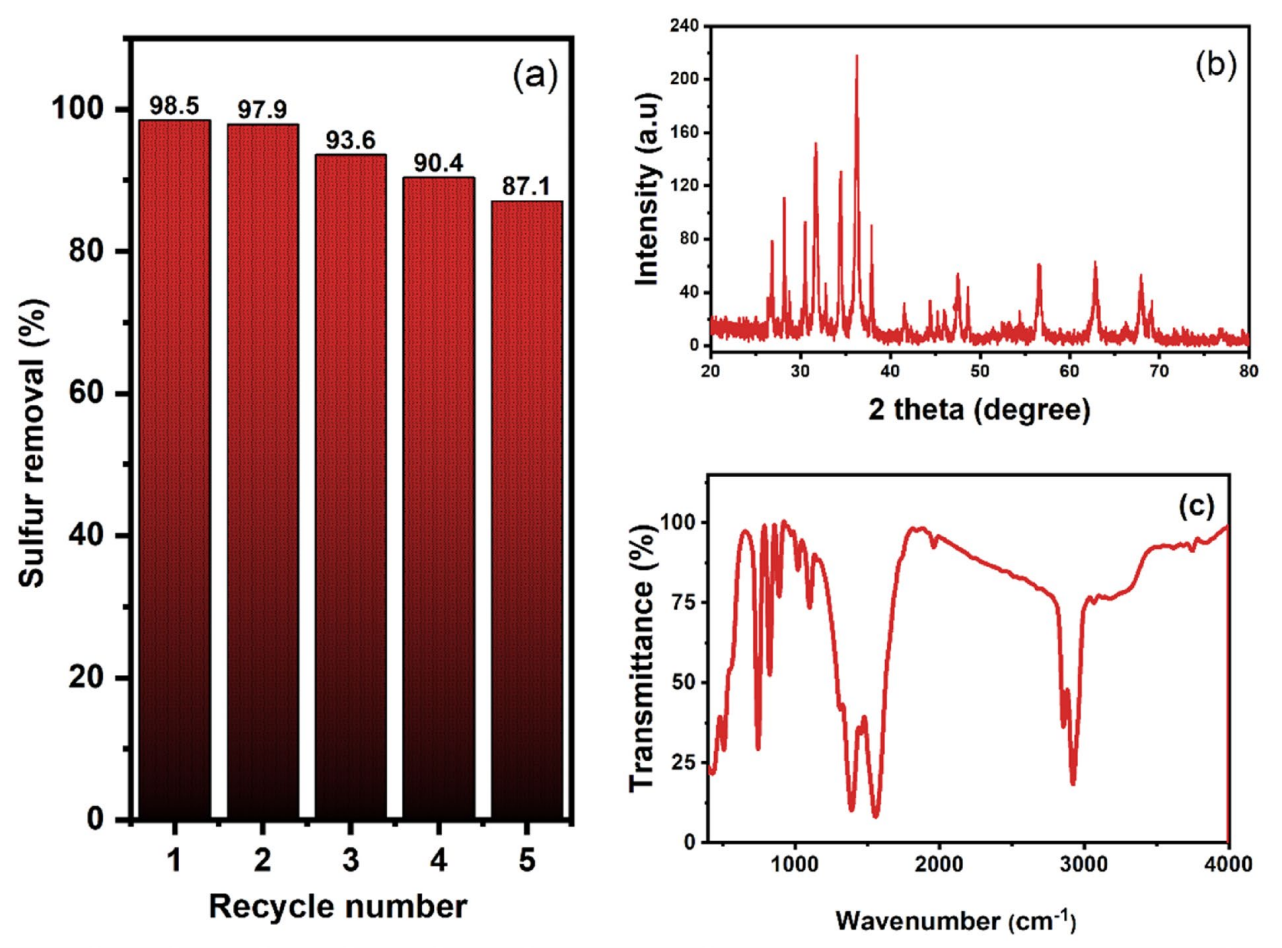
(A) The reusability of the catalyst; (B) XRD; and (C) FTIR of reused catalyst.

Assessing the practical applicability of ZnO-Zn-MOF/BNC, the reusability and stability were crucial considerations. The catalyst’s stability was evaluated by conducting a reusability test under optimal conditions. Following each cycle, the separated catalyst underwent regeneration through ethanol washing and oven drying at 120 °C overnight. Subsequently, the regenerated catalyst was employed in subsequent ODS cycles. The catalytic activity of the catalyst after six runs revealed only about 11.4% reduction in activity (Fig. 13A).

This steady state of the regenerated ZnO-Zn-MOF/BNC was further investigated by XRD and FTIR analyses (Fig. 13B & C), which initially revealed no significant changes in crystallinity or functional group composition before and after reuse. These findings collectively confirm that the catalyst maintains its most structural integrity, where its pore architecture plays a crucial role in maintaining high recyclability and sustained catalytic performance.

For evaluating the effectiveness of the synthesized ZnO-Zn-MOF/BNC catalyst, its performance in desulfurization was compared with other catalysts in the literature (Table 2). Under optimum conditions, The ZnO-Zn-MOF/BNC catalyst removed 98.5% of sulfur from real diesel fuel containing 1200 ppm sulfur under optimized conditions, representing one of the highest reported for real fuel systems utilizing eco-friendly and benign oxidative desulfurization (ODS) conditions.

**Table 1:** Physicochemical characterization of diesel oil before and after EODS treatment at optimum conditions.

Parameter	Diesel oil before EODS	Diesel oil after ODS	Method
Sulfur content (ppm)	1200	18.1	
Density @ 15.56 °C	0.8259	0.8212	ASTM D-1298
Specific gravity	0.8267	0.8220	ASTM D-1298
API gravity	39.67	40.65	ASTM D-1298
Aniline Point (^o^C)	79	82	ASTM D-611
Refractive index @ 20 °C	1.45974	1.45647	ASTM D-1218

**Table 2:** Comparison of catalyst ZnO-Zn-MOF/BNC (O) with other studies on the ODS reaction of real fuel.

Catalyst		Sulfur removal (%)	Condition	
ZnO-Zn-MOF/BNC	Diesel fuel (1200 ppm)	98.5	T = 70 °C, t = 60 min, O (H_2_O_2_)/S = 8, Cat. Wt.= 200 mg, solvent: diesel oil = 1.5:1	*This study*
PMB-3	Diesel oil (720 ppm)	98.3	T = 45 °C, t = 45 min, O (H_2_O_2_/CH_3_COOH), Wt. of cat. of 2 g/L, solvent= ml	^10^
MoO_3_/Al_2_O_3_,	Hydrotreated middle distillate (445ppm)		T = 75 °C, t = min, O (H_2_O_2_)/S = 20, cat. Wt.= 5 wt%, solvent= ml	^62^
(MoO_3_/SG)	Commercial diesel (590 ppm)	99.9	T = 45 °C, t = 90 min, O (H_2_O_2_)/S = 8, M catalyst/Voil of 0.1 g/mL solvent= ml	^63^
Mo@COMOC-4	Commercial diesel (939 ppm)	74	T = 70 °C, t = 50 h, O (TBHP/S = 12	^64^
W40 − MCM-41		63	T = 60 °C, t = 120 min, H2O2/S (M) of 4:1, cat. Wt.= 1 wt%, solvent= ml	^65^
Mo@COMOC-4	Original diesel (639 ppm)	74	T = 70 °C, t = min, O (TBHP)/S = 12, solvent= ml	^64^
Boron nitride (BN)	Diesel fuel (8040 ppm)	72.4	T = 71 °C, t = 113 min, O (H_2_O_2_)/S = 10.2, Cat. Wt.= 0.36 gm, solvent= ml	^66^

Compared to commercial catalysts like MoO₃/SG, which achieved 99.9% sulfur removal from commercial diesel with 590 ppm sulfur at 45 °C for 90 min, the ZnO-Zn-MOF/BNC catalyst showed comparable performance (98.5%) with more than two-folds of the initial sulfur content and in only two-thirds of the reaction time. Also outperforming Mo@COMOC-4, which after 50 h of reaction time only managed 74% sulfur removal from commercially available diesel containing 939 ppm sulfur. Boron nitride (BN) suffered from low efficiency delivering only 72.4% sulfur removal due to high operating temperature and sulfur content. Our results differ from PMB-3, which achieved 98.3% desulfurization from 720 ppm sulfur content diesel, by requiring 2 g/L catalyst loading and an entirely different oxidant system.

The current study encountered several limitations mainly because of its multidisciplinary work-scheme, which made the design didn’t include some comprehensive investigations. The micro- or nanostructure of the synthesized ZnO-Zn-MOF/BNC may deserve advanced imaging utilizing cross-section SEM to assure the homogenous synthesis of the MOF throughout the BNC tridimensional nanofibrous matrix. In addition, the structural integrity and thermal stability of the catalyst after regeneration have to be inspected excessively. Deeper thermodynamic analysis should be carried out for realizing how the ODS process eventuates and figuring out the factors interfering its phases. Considering the promising performance of the ZnO-Zn-MOF/BNC in the current work, the next part will be planned for clarifying these blind spots before scaling up to a pilot model.

## Conclusions

We believe that replacing conventional industrial materials with green materials that have comparable functional efficiency will have milder environmental impacts even if they enter the ecosystem in large quantities. In this study, a sustainable ZnO-Zn-MOF/bacterial nanocellulose (BNC) composite was successfully synthesized using a green one-pot solvothermal approach. The comprehensive characterization confirmed the formation of a well-structured hybrid material with enhanced physicochemical properties. We surmise that the incorporation of BNC significantly improved the overall composite crystallinity, dispersion, pore connectivity, stability, in addition to the ecological footprints; proving the efficiency of the solvothermal route for fabricating such composites. The catalytic activity of the ZnO-Zn-MOF/BNC nanocomposite was evaluated in the extractive oxidative desulfurization (EODS) of real diesel fuel. Under the optimum conditions, the catalyst achieved a sulfur removal efficiency of 98.5% within 60 min without compromising its structural or catalytic integrity even after five successive cycles. Our future work will focus on evaluating the ODS performance of the ZnO-Zn-MOF/BNC in continuous-flow systems and pilot-scale operations supported by keener structural and thermodynamic investigations to further validate its scalability and operational viability in the industrial desulfurization processes.

## Data Availability

The datasets used and/or analyzed during the current study are available from the corresponding author on reasonable request.
